# A Nationwide Analysis of Fluid Resuscitation Outcomes in Patients With Acute Pancreatitis

**DOI:** 10.7759/cureus.50182

**Published:** 2023-12-08

**Authors:** Julton Tomanguillo, Lauren Searls, Frank H Annie, Suzanne Kemper, Kerry Drabish, Vishnu Naravadi

**Affiliations:** 1 Internal Medicine, Charleston Area Medical Center (CAMC) Institute for Academic Medicine, Charleston, USA; 2 Cardiology, Charleston Area Medical Center (CAMC) Institute for Academic Medicine, Charleston, USA; 3 Outcomes Research, Charleston Area Medical Center (CAMC) Health Education and Research Institute, Charleston, USA; 4 Research, Charleston Area Medical Center (CAMC) Institute for Academic Medicine, Charleston, USA; 5 Gastroenterology, Charleston Area Medical Center (CAMC) Institute for Academic Medicine, Charleston, USA

**Keywords:** treatment outcomes, resuscitation, pancreatitis, infusion intravenous, fluid therapy

## Abstract

Background: Traditionally, fluid resuscitation has been the foundation of effective acute pancreatitis (AP) treatment. Experts advocate for aggressive intravenous fluid (IVF) resuscitation, especially within the first 24 hours. Research suggests limited efficacy of this approach; in fact, some studies show there may be a risk of increased complications. The aim of this study was to assess outcomes of aggressive IVF resuscitation in patients with AP.

Methods: We queried the TriNetX Research Network (Cambridge, Massachusetts, United States) for patients admitted between January 1, 2010, and December 31, 2020, with a diagnosis of AP and who had received IVF on admission for at least 24 hours. We compared two cohorts; cohort 1 consisted of patients receiving aggressive IVF resuscitation (>3 ml/kg/hr), and cohort 2 was comprised of patients receiving non-aggressive (≤1.5 ml/kg/hr) IVF resuscitation. We compared mortality during index hospitalization, hospital length of stay (HLS), mechanical ventilation rates, acute kidney injury (AKI), and severe sepsis between the cohorts with propensity scoring matched (PSM) pairs of patients. A sub-analysis of patients with severe AP was performed.

Results: After comparing the two well-matched PSM cohorts (3,680/3,680), we found no significant differences in mortality, HLS, mechanical ventilation rates, AKI, or severe sepsis. We found similar results after conducting the sub-analysis of patients with severe pancreatitis.

Conclusions: We found no significant differences in mortality and HLS when comparing rates of IVF resuscitation.

## Introduction

Acute pancreatitis (AP) is a severe inflammatory process of the pancreas characterized by intractable upper abdominal pain, increase of serum lipase levels, and/or inflammatory changes noted in imaging. This pathology is associated with significant increase of morbidity, mortality, and healthcare economic burden [1-4]. As high as 15% of the patients with AP died, and in those with associated organ failure, which accounts for one third of all AP, mortality can increase up to 30% [5]. An estimated 210,000 hospital admissions for AP occur annually, with a total annual cost of approximately $2.2 billion [6]. These factors make the treatment of AP a priority with evolving guidelines for care. AP is characterized by a systemic inflammatory response that leads to capillary leak, decrease intravascular volume, third spacing, organ hypoperfusion, and untimely organ failure and death if not treated appropriately [7]. There have not been any new therapeutic advances in the treatment of AP; however, intravenous fluids (IVF) have been shown to counteract some of the detrimental effects of capillary leak by increasing intravascular volume and improving organ perfusion [2]. Aggressive IVF resuscitation has become a topic of interest due to concerns that may cause harm when compared to non-aggressive IVF resuscitation. Mortality, acute kidney injury (AKI), acute respiratory distress syndrome, persistent organ failure, and pancreatic necrosis have been targets of recent studies investigating IVF administration [7-9]. With the growing concern of detrimental fluid overload, there is a need for further investigation. The aim of this study is to compare 30-day mortality and hospital length of stay (HLS) among patients with AP who received aggressive IVF resuscitation compared to those who received non-aggressive IVF resuscitation within the first 24 hours of presentation to the emergency room.

## Materials and methods

We queried the TriNetX Research Network (Cambridge, Massachusetts, United States) for patients admitted between January 1, 2010, and December 31, 2020, with a diagnosis of AP and who had received IVF on admission for at least 24 hours [1]. We compared two cohorts; cohort 1 consisted of patients receiving aggressive IVF resuscitation (>3 ml/kg/hr), and cohort 2 was comprised of patients receiving non-aggressive (≤1.5 ml/kg/hr) IVF resuscitation. We compared mortality during index hospitalization, HLS, mechanical ventilation rates, AKI, and severe sepsis between the cohorts with propensity scoring matched (PSM) pairs of patients. A sub-analysis of patients with severe AP was performed [1].

This study was conducted in Charleston Area Medical Center (CAMC), Charleston, West Virginia, United States, and utilized the TriNetX Research Network, a longitudinal federal database of electronic medical records. The extensive database contains deidentified information for 110 million patients across 67 healthcare organizations in the United States, comprising hospital, primary care, and specialty care organizations providing both outpatient and inpatient services. The data features comprehensive details such as demographics, diagnoses based on International Classification of Diseases (ICD) codes, and laboratory tests conducted alongside utilization of healthcare resources including procedures and medications administered. Recent clinical studies have used the TriNetX platform to investigate various areas requiring answers ranging from patient health outcomes to prescription-administration practices.

For categorical variables, the TriNetX platform calculates descriptive statistics as frequencies with percentages; for continuous measures, means±standard deviations are computed. The platform uses 1:1 PSM using a logistic regression for scores of the different metric used in the analysis. The PSM uses the Python libraries (NumPy and Sklearn).

## Results

**Figure 1:**
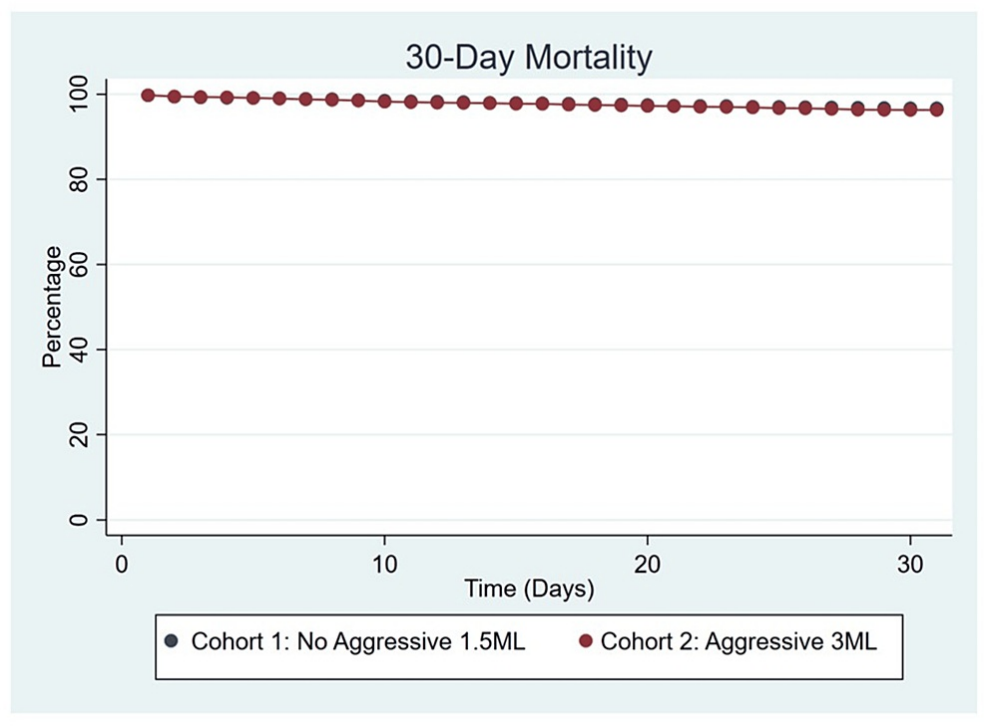
Non-aggressive IVF resuscitation versus aggressive IVF resuscitation 30-day mortality IVF: intravenous fluid

A total of 10,400 patients were initially included in the study. Prior to PSM analysis, patients who received non-aggressive IVF resuscitation (N=6,718) were younger (52.6±19.1, p=0.01) and had a higher rate of diabetes (25.1% vs. 22.2%, p<0.001) and chronic kidney disease (8.5% vs. 7.5%, p=0.07) when compared to the aggressive IVF resuscitation cohort (N=3,682) (Table 1). Two well-matched cohorts were created (3,680/3,680) based on age, race, sex, and key comorbidities such as diabetes, coronary artery disease, chronic kidney disease, and heart failure. Analysis showed no significant difference between 30-day mortality (2.8% vs. 3.0%, p=0.58), HLS (2.4 days vs. 2.39 days, p=0.97), mechanical ventilation rates (1.5% vs. 1.7%, p=0.35), AKI (5.9% vs. 5.4%, p=0.31), and severe sepsis (4.3% vs. 3.5%, p=0.10) (Table 1 and Table 2). All endpoints were confirmed with a log-rank test and hazard ratio models. A sub-analysis was performed with the PSM sample (N=7,360). Two well-matched sub-cohorts were created (157/157) based on the severity of the pancreatitis and the approach to fluid resuscitation. A well-validated Bedside Index of Severity in Acute Pancreatitis (BISAP) score was used to stratify the patients based on severity of pancreatitis. A score ≥3 indicated severe pancreatitis (Figure 1). Analysis showed no significant difference between non-severe pancreatitis patients who received aggressive fluid resuscitation and patients with severe pancreatitis who received non-aggressive fluid resuscitation: 30-day mortality (24.2% vs. 24.8%, p=0.89), HLS (29 days vs. 32 days, p=0.71), mechanically ventilation rates (12.7% vs. 12.7%, p=1.00), AKI (23.6% vs. 22.9%, p=0.90), and severe sepsis (27.4% vs. 27.4%, p=1.00) (Table 2).

**Table 1:** Baseline characteristics CAD: coronary artery disease; CKD: chronic kidney disease; SD: standard deviation The data has been represented as N, %, mean±SD, and p-value considered significant at 0.05.

	Unmatched cohorts				Propensity-matched cohorts			
Baseline characteristics	Non-aggressive fluid resuscitation (N=6,718)	Aggressive fluid resuscitation (N=3,682)	p-value	Standardized mean difference	Non-aggressive fluid resuscitation (N=3,680)	Aggressive fluid resuscitation (N=3,680)	p-value	Standardized mean difference
Age at index	52.6±19.1	53.5±18.7	0.01	0.05	53.3±18.8	53.5±18.7	0.74	0.01
White	83.1%	82.1%	0.20	0.03	83.3%	82.15%	0.21	0.03
Female	55.0%	55.4%	0.64	0.01	55.8%	55.44%	0.76	0.01
Male	45.0%	44.5%	0.62	0.01	44.2%	44.54%	0.78	0.01
Black/African American	10.4%	10.4%	0.99	0.00	9.7%	10.44%	0.31	0.02
Unknown race	4.9%	6.2%	<0.001	0.06	6.0%	6.20%	0.66	0.01
Diabetes	25.1%	22.2%	0.00	0.07	21.7%	22.17%	0.59	0.01
CAD	10.4%	10.6%	0.76	0.01	10.1%	10.57%	0.54	0.01
CKD	8.5%	7.5%	0.07	0.04	7.1%	7.50%	0.47	0.02
Heart failure	0.2%	0.3%	0.63	0.01	0.3%	0.27%	1.00	0.00

**Table 2:** Study outcomes IVF: intravenous fluid; SD: standard deviation The data has been represented as N, %, mean±SD, and p-value considered significant at 0.05.

Outcomes	Non-aggressive IVF resuscitation (N=3,680)	Aggressive IVF resuscitation (N=3,680)	p-value
30-day mortality	3.0%	2.8%	0.58
Hospital length of stay	2.39 days	2.4 days	0.97
Mechanical ventilation rates	1.7%	1.5%	0.35
Acute kidney injury	5.4%	5.9%	0.31
Severe sepsis	3.5%	4.3%	0.10
Sub-analysis: severity of pancreatitis			
Outcomes	Non-aggressive IVF resuscitation (N=3,680)	Aggressive IVF resuscitation (N=3,680)	p-value
30-day mortality	24.8%	24.2%	0.89
Hospital length of stay	32 days	29 days	0.71
Mechanical ventilation rates	12.7%	12.7%	1.00
Acute kidney injury	22.9%	23.6%	0.90
Severe sepsis	27.4%	27.4%	1.00

## Discussion

The pathophysiology of AP is mainly centered on the disruption of the pancreatic microcirculation. The injury to the pancreatic acinar cell leads to an overwhelming production of cytokines and vasoactive peptides [10-14]. The endothelial dysfunction, vasospasm, and microthrombi formation because of these mediators lead to pancreatic necrosis and hemodynamical instability [14-16]. These detrimental effects are counteracted by IVF administration which improves and maintains the microvascular pancreatic blood perfusion [16-18].

Multiple guidelines for the last 20 years have recommended aggressive IVF resuscitation as the cornerstone treatment of AP [5-10,19-21]. However, bench and clinical studies that support these recommendations may be affected due to reverse causation bias. This bias indicates that the outcomes caused the exposure instead of the contrary. Patients with AP usually develop third spacing, which leads to decreased intravascular volume, decreased renal perfusion, oliguria, hemoconcentration, and hypotension; therefore, patients are treated with aggressive IVF [2].

There is raising concern of aggressive IVF administration and its potential detrimental outcomes in patients with AP. Ye et al. reported that patients who received aggressive IVF resuscitation, defined as >4 L in the first 24 hours, had an increased incidence of AKI (53.12% vs. 25.65%, p=0.008) and utilization of renal replacement therapy (40.63% vs. 24.36%, p=0.108) [8]. Similarly, de-Madaria et al. reported that patients who received more than 4 L were associated with persistent organ failure, respiratory insufficiency, and AKI [9]. A recent meta-analysis by Gad and Simons-Linares reported that patients who received aggressive IVF had a higher risk for AKI and respiratory failure, with no improvement in mortality [7].

The literature surrounding IVF resuscitation in AP currently has limitations including the lack of a clear definition of aggressive IVF resuscitation, as well as lacking data with stratification of AP patients by severity. A definition for IVF resuscitation rates was proposed by Garg and Mahapatra, suggesting a defined aggressive IVF resuscitation as a fluid rate of 3 ml/kg/h and 1.5 ml/kg/h as non-aggressive [22]. Our study identified patients with AP and classified non-aggressive IVF as ≤1.5 ml/kg/hr (non-aggressive IVF cohort) and those who received aggressive IVF >3 ml/kg/hr (aggressive IVF cohort). A sub-analysis of severe pancreatitis was also performed, with severe AP defined as BISAP score >3. The BISAP score is a validated scoring tool that helps to identify the severity of pancreatitis and the risk of developing necrotic pancreatitis. To assess the severity of the pancreatitis, this score assesses the level of blood urea nitrogen (BUN), mental status, age, and pleural effusion [22].

Our study provided additional data to suggest aggressive IVF resuscitation may not be beneficial in patients with AP. The lack of benefit on HLS and mortality suggests that aggressive IVF may not be the right approach in hospitalized patients, even in cases of severe AP. We also analyzed AKI development, severe sepsis, and need for mechanical ventilation, with no statistically significant difference between the aggressive IVF and non-aggressive IVF cohorts. Recently, the WATERFALL Trial compared the outcomes of moderate (defined as 10 ml/kg bolus in hypovolemic patient or no bolus in euvolemic patients, followed by 1.5 ml/hr) versus aggressive IVF resuscitation (defined as 20 ml/kg bolus followed by 3 ml/kg/hr) in AP patients. This randomized clinical trial shows some evidence that aggressive IVF resuscitation has more detrimental outcomes compared to other IVF approaches; a higher incidence of fluid overload (20.5% vs. 6.3%) and prolong HLS (6±2 vs. 5±2) were found in this study.

In this nationwide analysis with a large sample size, patients who received aggressive IVF within the first 24 hours of admission did not show a significant association with improvement of HLS or mortality. It has been hypothesized that aggressive IVF resuscitation, especially in severe pancreatitis, leads to increased vascular leak and subsequent congestion, sequestration, tissue hypoxia, and ultimately worsening of organ dysfunction [10]. Our study provides some additional data to suggest that aggressive IVF may not have benefit to patients with AP. We utilized a large, nationwide database comprising 67 healthcare facilities (TriNetX), allowing us to include a large sample size with a diverse array of patients. Additionally, the use of PSM analysis created a balanced dataset between cohorts, helping control and reduce measurable confounders.

We identified possible limitations to this study. We used a retrospective design, which introduces potential selection bias and the inability to assess incidence. Also, we used deidentified data, which may decrease the recognition of confounding variables that could affect treatment decision-making processes specific to each patient. In addition, we recognize that patients may have presented to the emergency department in different stages of pancreatitis presentation, leading to varying IVF rates and severity of disease. Finally, we did not consider confounding variables impacting disease processes and healing, such as the possibility of enteral nutrition or the type of fluid used for IVF resuscitation.

## Conclusions

We found no significant differences in 30-day mortality or HLS between aggressive and non-aggressive IVF resuscitation, even in patients with severe pancreatitis on admission. Additionally, there were no statistically significant differences noted in AKI, severe sepsis, and need for mechanical ventilation. We would like to recommend an individualized, goal-directed rate of IVF resuscitation in patients admitted with AP.
